# Draft genome sequence of *Pediococcus acidilactici* 3G3 isolated from Philippine fermented pork

**DOI:** 10.1128/mra.01299-23

**Published:** 2024-03-25

**Authors:** Zachary B. Lara, Mia Beatriz C. Amoranto, Francisco B. Elegado, Leslie Michelle M. Dalmacio, Marilen Parungao Balolong

**Affiliations:** 1 Department of Biochemistry and Molecular Biology, College of Medicine, University of the Philippines, Manila, Philippines; 2 National Institute of Molecular Biology and Biotechnology, University of the Philippines Los Baños, Los Baños, Philippines; 3 Department of Biology, College of Arts and Sciences, University of the Philippines Manila, Manila, Philippines; Loyola University Chicago, Chicago, Illinois, USA

**Keywords:** probiotics, lactic acid bacteria, Philippines, fermented food, illumina, genomes, *Pediococcus acidilactici*

## Abstract

*Pediococcus acidilactici* is a well-studied fermentative bacterium reported to have potential probiotic properties. Here, we report the draft genome of *P. acidilactici* 3G3 previously isolated from *burong babi*, a Philippine fermented pork dish. The 3G3 draft genome has 1,886,498 bp, a GC content of 42.27%, and a completeness of 99.38%.

## ANNOUNCEMENT

*Pediococcus acidilactici* is a gram-positive, facultative anaerobe with strict homofermentative metabolism (1). Several *P. acidilactici* strains have been used in the food industry owing to their antimicrobial and potential probiotic properties. A previous study identified *P. acidilactici* 3G3, which was isolated from Philippine fermented pork, as a promising candidate for probiotic food applications (2). Furthermore, this strain significantly improved the safety and nutritional and sensory properties of *pindang damulag* (fermented carabeef) (3). Evaluating its safety and probiotic potential using whole-genome sequencing is essential for the development of novel functional food products and starter cultures. Herein, we report the draft genome of *P. acidilactici* 3G3.

*P. acidilactici* 3G3 was previously isolated from *burong babi*, a fermented pork dish, obtained from a wet market in Pampanga, Philippines (3). We obtained the strain from the collection of the Food and Feeds Laboratory of the National Institute of Molecular Biology and Biotechnology, University of the Philippines Los Baños (Laguna, Philippines). Briefly, 3G3 was grown in de Man, Rogosa, and Sharpe (HiMedia Laboratories, Germany) at 37°C for 24 h. Genomic DNA (gDNA) was extracted using the Wizard gDNA Purification Kit (Promega Corporation, WI, USA) according to the manufacturer’s protocol, with minor modifications (i.e., extended incubation time and air drying; use of cold isopropanol and ethanol for DNA precipitation and washing, respectively). Library preparation was performed using the TruSeq DNA Nano Kit (Illumina, USA), and sequencing was conducted using an Illumina MiSeq instrument (2 × 150 cycles) at the Philippine Genome Center (Quezon City, Philippines). A total of 2,994,562 reads, with an average length of 150 bp, were generated. Quality control was performed using fastp 0.23.2 (4). Contigs were assembled using Spades 3.15.5 (5), with the *--sc* and *--careful* presets specified—a combination producing the least amount of mis-assemblies. Scaffold quality was assessed using Quast 5.2.0 (6) and CheckM 1.1.6 (7), while predicted genes were annotated using the NCBI Prokaryotic Genome Annotation Pipeline 6.6 (8). Phylogenetic analysis was performed using the Microbial Genomes Atlas web server (9), while average nucleotide identity (ANI) was calculated using FastANI 1.3.3 (10). Default settings were used for all software unless otherwise specified.

We present the first genome report of *P. acidilactici* isolated in the Philippines. The 3G3 genome contains 1,886,498 bp (*N*50 = 392,558 bp, coverage = 234.9×) in 14 contigs, a GC content of 42.27%, and completeness of 99.38%. The strain shared an average nucleotide identity of 98.67% with *P. acidilactici* FDAARGOS_1007 (NZ_CP066046.1), the closest related strain identified to date. A total of 1,812 predicted coding sequences, 3 rRNAs, and 39 tRNAs were annotated.

## Data Availability

The draft genome assembly and raw reads are deposited in NCBI GenBank under the accession numbers GCF_031032815 and SRS18543368, respectively.
